# Minor Cause—Major Effect: A Novel Mode of Control of Bistable Gene Expression

**DOI:** 10.1371/journal.pgen.1005229

**Published:** 2015-06-25

**Authors:** Jan Kampf, Jörg Stülke

**Affiliations:** Department of General Microbiology, Georg-August-Universität Göttingen, Göttingen, Germany; Indiana University, UNITED STATES

For a long time, bacterial populations were regarded as being uniform with respect to the physiology and morphology of the individual cells. However, a variety of different sub-populations may coexist in seemingly uniform populations. This was first observed for the formation of genetically competent cells of the Gram-positive model organism *Bacillus subtilis*. Genetic competence is the ability to take up and incorporate free DNA from the environment into a cell. This allows the cell to acquire new properties, but comes at the risk that the new DNA may also cause harm to the receiving cell. Therefore, it is not surprising that, even under conditions that trigger the development of competence, only about 10% of a population enters the competent state. The phenomenon that underlies this phenotypic heterogeneity at the level of gene expression is referred to as bistability or bistable gene expression [1,2]. The molecular triggers that cause the separation into distinct subpopulations are not yet fully explored. Importantly, a major factor in bistability is the existence of positive or negative feedback loops [1,3,4]. In this issue of *PLOS Genetics*, Gamba et al. [5] have identified yet another feedback loop that controls bistable expression of competence genes in *B*. *subtilis*.

The best-studied examples of bistable gene expression are: the entry to sporulation, the development of genetic competence, and the choice between motility and biofilm formation; all in *B*. *subtilis*. These examples also helped to uncover basic principles in the design of the feedback loops resulting in heterogeneity, and of the determining molecular events. At the mechanistic level, bistability is caused by a signal (a protein or a molecule) that is normally present at low concentration, but which is strongly amplified once a certain threshold has been exceeded. This threshold can be reached in individual cells due to the intrinsic stochastic variability in gene expression, also referred to as noise [6]. As the primary event in bistability, the concentration or activity of a key regulator of the network must be subject to stochastic regulation. This regulation may occur by controlling the cellular concentration of the protein by proteolysis, by regulatory protein-protein interactions or by covalent modification. Indeed, all these mechanisms have been demonstrated as being decisive for bistable gene expression in *B*. *subtilis* (see Table 1) [7].

**Table 1:** Molecular mechanisms behind bistable gene expression in *B*. *subtilis*.

Regulation level	Example
mRNA stability	*comK* mRNA by Kre [5], *sinR* mRNA by RNaseY
Protein stability	ComK by ClpCP [9], SlrR (autocleavage)
Protein-protein interaction	SinR-SlrR, SinR-SinI
Post-translational modification	Spo0A phosphorylation
Position effect in a long operon	Motility development due to sigD gene position

Gamba et al. [5] have now used transposon mutagenesis to identify a novel player in bistable expression of competence genes. Importantly, they also unravel a novel level of control of bistability—by controlling the stability of the mRNA of a key transcription factor. The analysis of bistable gene expression is often hampered by the complexity of the involved regulatory elements. This is also the case for the control of the genes required for the uptake and integration of foreign DNA in *B*. *subtilis*, collectively called the competence genes. The expression of these genes is activated by the transcription factor ComK. The expression of the *comK* gene is regulated by at least six different transcription regulators, including ComK itself (see http://subtiwiki.uni-goettingen.de/wiki/index.php/ComK for an overview [8]). Moreover, the ComK protein is degraded by the ClpCP protease at low cell densities [9]. This complex control results in bistable expression of ComK, i.e., one portion of a population growing in competence medium expresses ComK, whereas the other portion does not. Gamba and colleagues have now designed a clever screening system to uncover novel factors that are involved in the control of bistable expression of ComK. For this purpose, they expressed the *comK* gene under the control of a promoter (*comG*) that is exclusively controlled by ComK in a strain unable to degrade the ComK protein. Interestingly, even this minimal expression system for ComK still allows bistable expression of ComK and of genetic competence [10]. Transposon mutagenesis of this strain and a screen for mutants with high competence promoter activity and expression of competence genes in the majority of cells resulted in the identification of Kre (ComK repressor), a novel factor in the control of bistable gene expression.

In a set of elegant experiments, the authors demonstrate that the loss of Kre results in overexpression of ComK, uniform expression of competence genes, and increased genetic competence. In contrast, overexpression of Kre resulted in the opposite phenotypes. The function of Kre could be attributed to the control of ComK mRNA stability, i.e., in a *kre* mutant, the half-life of the *comK* mRNA increases. Thus, Kre is a novel factor that affects mRNA stability, and therefore an addition to the network of proteins implicated in RNA degradation in *B*. *subtilis*. It is tempting to speculate that Kre might modify the activity of one of the endo- and exoribonucleases that interact to achieve coordinated RNA degradation and processing in *B*. *subtilis* [11]. Strikingly, the relation between Kre and ComK is not limited to the control of *comK* mRNA stability but also involves the repression of *kre* transcription by ComK in competent cells (see Fig 1). Thus, Kre and ComK form a double negative feedback loop that is characteristic for the control of bistable gene expression. The functional significance of the link between Kre and ComK is further supported by the observation that the Kre protein is present only in bacteria that also contain ComK.

**Fig 1:**
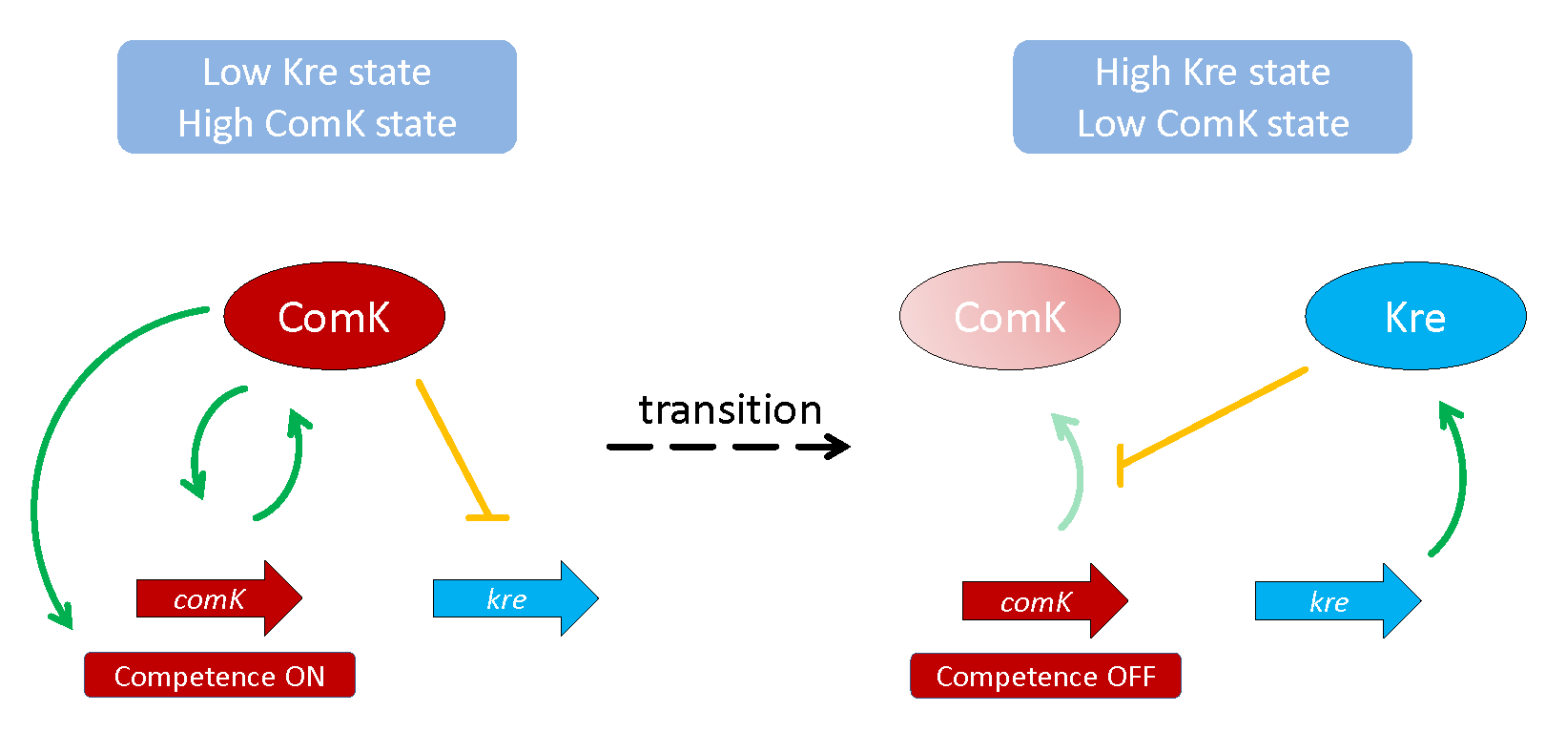
Feedback loops that control the bistable gene expression of genetic competence in *B*. *subtilis*. If Kre is low, the *comK* mRNA and, thus, ComK protein can accumulate, resulting in the expression of competence genes. In contrast, high activity of Kre results in degradation of *comK* mRNA, and the cells do not enter the competence state.

The fact that a key regulator of a bistable expression system is controlled at the level of mRNA stability is not unprecedented: recently, it has been demonstrated that the mRNA for the master regulator of biofilm formation, SinR, is subject to control by RNase Y [12]. Moreover, RNA polymerase processivity or mRNA turnover of the very long *B*. *subtilis* operon encoding motility genes including the sigma factor gene *sigD* were also shown to be the decisive factors for stochastic expression of motility genes at the level of single cells [13]. The study of Gamba et al. [5] reinforces the implication of mRNA stability for bistable gene expression. This work opens a window to novel mechanisms of the control of bistable gene expression and paves the way to their discovery.

## References

[1] Smits WK, Kuipers OP, Veening JW (2006) Phenotypic variation in bacteria: the role of feedback regulation. Nat Rev Microbiol 4: 259–271. 16541134 10.1038/nrmicro1381

[2] Dubnau D, Losick R (2006) Bistability in bacteria. Mol Microbiol 61: 564–572. 16879639 10.1111/j.1365-2958.2006.05249.x

[3] Tiwari A, Ray JC, Narula J, Igoshin OA (2011) Bistable responses in bacterial genetic networks: designs and dynamical consequences. Math Biosci 231: 76–89. 10.1016/j.mbs.2011.03.004 21385588 PMC3095517

[4] Casadesus J, Low DA (2013) Programmed heterogeneity: epigenetic mechanisms in bacteria. J Biol Chem 288: 13929–13935. 10.1074/jbc.R113.472274 23592777 PMC3656251

[5] Gamba P, Jonker MJ, Hamoen LW (2015) A novel feedback loop that controls bimodal expression of genetic competence. PLoS Genet 11(6): e1005047 10.1371/journal.pgen.1005047 26110430 PMC4482431

[6] Qian H (2012) Cooperativity in cellular biochemical processes: noise-enhanced sensitivity, fluctuating enzyme, bistability with nonlinear feedback, and other mechanisms for sigmoidal response. Annu Rev Biophys 41: 179–204. 10.1146/annurev-biophys-050511-102240 22404682

[7] Veening JW, Smits WK, Kuipers OP (2008) Bistability, epigenetics, and bet-hedging in bacteria. Annu Rev Microbiol 62: 193–210. 10.1146/annurev.micro.62.081307.163002 18537474

[8] Michna RH, Commichau FM, Tödter D, Zschiedrich C, Stülke J (2014) SubtiWiki—a database for the model organism *Bacillus subtilis* that links pathway, interaction and expression information. Nucleic Acids Res 42: D692–D698. 10.1093/nar/gkt1002 24178028 PMC3965029

[9] Turgay K, Hahn J, Burghoorn J, Dubnau D (1998) Competence in *Bacillus subtilis* is controlled by regulated proteolysis of a transcription factor. EMBO J 17: 6730–6738. 9890793 10.1093/emboj/17.22.6730 PMC1171018

[10] Smits WK, Eschevins CC, Susanna KA, Bron S, Kuipers OP, Hamoen LW (2005) Stripping *Bacillus*: ComK auto-stimulation is responsible for the bistable response in competence development. Mol Microbiol 56: 604–614. 15819618 10.1111/j.1365-2958.2005.04488.x

[11] Lehnik-Habrink M, Mäder U, Lewis RJ, Stülke J (2012) RNA degradation in *Bacillus subtilis*: an interplay of essential endo- and exoribonucleases. Mol Microbiol 84: 1005–1017. 10.1111/j.1365-2958.2012.08072.x 22568516

[12] Lehnik-Habrink M, Schaffer M, Mäder U, Diethmaier C, Herzberg C, Stülke J (2011) RNA processing in *Bacillus subtilis*: identification of targets of the essential RNase Y. Mol Microbiol 81: 1459–1473. 10.1111/j.1365-2958.2011.07777.x 21815947

[13] Cozy LM, Cairns DB (2010) Gene position in a long operon governs motility development in *Bacillus subtilis* . Mol Microbiol 76: 273–285. 10.1111/j.1365-2958.2010.07112.x 20233303 PMC2911795

